# Device-Associated Hospital-Acquired Infections: Does Active Surveillance With Bundle Care Offer a Pathway to Minimize Them?

**DOI:** 10.7759/cureus.19331

**Published:** 2021-11-07

**Authors:** Vithiya Ganesan, Raja Sundaramurthy, Rajendran Thiruvanamalai, Vijay Anand Sivakumar, Sridhurga Udayasankar, Ramesh Arunagiri, Jhansi Charles, Sunil Kumar Chavan, Yuvaraj Balan, Varatharajan Sakthivadivel

**Affiliations:** 1 Department of Microbiology, Velammal Medical College Hospital and Research Institute, Madurai, IND; 2 Department of Microbiology, All India Institute of Medical Sciences - Bibinagar, Hyderabad, IND; 3 Department of Anaesthesiology, Velammal Medical College Hospital and Research Institute, Madurai, IND; 4 Department of Paediatrics, Velammal Medical College Hospital and Research Institute, Madurai, IND; 5 Department of Biochemistry, All India Institute of Medical Sciences - Bibinagar, Hyderabad, IND; 6 Department of Medicine, All India Institute of Medical Sciences - Bibinagar, Hyderabad, IND

**Keywords:** bundle care, device associated infections, central line, ventilator, urinary catheter

## Abstract

Background and objective

The prevalence of hospital-acquired infections (HAIs) is underreported in developing nations due to a lack of systematic active surveillance. This study reports the burden of device-associated HAIs (DA-HAIs) based on two years of active surveillance with in situ bundle care in closed intensive care units (ICUs) of a tertiary care hospital.

Materials and methods

A prospective surveillance study was carried out in 140-bedded ICUs (2,100-bed hospital) of a tertiary care private medical college hospital. Daily active surveillance for catheter-associated urinary tract infection (CAUTI), ventilator-associated event (VAE), and central line-associated bloodstream infection (CLABSI) was done by trained infection control nurses (ICNs) along with quality champion nurses with HAI surveillance forms with bundle care auditing, which was attached to the case sheets of all patients on devices. The surveillance definitions of DA-HAIs were adapted from the Centers for Disease Control and Prevention (CDC)’s National Healthcare Safety Network (CDC-NHSN) 2017 surveillance criteria. Data were analyzed at the end of every month to generate the cumulative device-associated infection (DAI) rates and device utilization ratio (DUR). These data were compared with NHSN and International Nosocomial Infection Control Consortium (INICC) - India HAI rates and communicated to corresponding ICUs and also presented at the hospital infection control committee (HICC) meeting.

Results

The surveillance data were reported over 71,877 patient days during the study period. The DUR of urinary catheters, ventilator, and central line were 0.53, 0.16, and 0.22, respectively. CAUTI, VAE, and CLABSI rates were 0.97, 10.5, and 0.43 per 1,000 device days, respectively. Among 166 DA-HAIs reported, 182 pathogens were identified. *Klebsiella pneumoniae* was the most common organism isolated, accounting for 37.4% of all DA-HAI cases, followed by *Acinetobacter baumanii (*30.8%). Most of the Gram-negative organisms were carbapenem-resistant (153/175; 87.4%). Vancomycin resistance rate in *Enterococcus* was 28.5% (2/7).

Conclusion

DUR and CAUTI, VAE, CLABSI rates were less/on par with the benchmarks of INICC and CDC-NHSN in almost all ICUs of our tertiary care unit. Gram-negative pathogen with 87.4% carbapenem resistance worsened the scenario. Proper active surveillance with bundle care and training by ICNs made a significant difference in all DA-HAI rates, especially VAE, which decreased to 10.5 from 23.6 per 1,000 ventilator days. Sustained active surveillance of HAI and bundle care auditing by a trained infection prevention team with a stringent antibiotic policy are the need of the hour to combat DAIs.

## Introduction

The Centers for Disease Control and Prevention (CDC) defines healthcare-associated infections (HAIs) as complications or infections secondary to either device implantation or surgery [1]. HAIs are associated with increased mortality, morbidity, and significant economic burden [2,3]. Device-associated hospital-acquired infections (DA-HAIs) constitute the majority of HAIs in intensive care units (ICUs) [4].

Active surveillance for HAI by a trained, designated, and unbiased team is the more efficient method when compared to passive surveillance (self HAI reporting by treating physicians) to know the exact burden and also to take proper preventive measures [5]. The prevalence of HAIs is underreported in developing nations due to a lack of systematic active surveillance [5].

This study reports a two-year active surveillance data of DA-HAIs and device utilization ratio (DUR) with in situ bundle care with their organisms and their antimicrobial profile in the ICUs of a tertiary care hospital in comparison with the CDC’s National Healthcare Safety Network (CDC-NHSN) and International Nosocomial Infection Control Consortium (INICC) - India HAI rates.

This article was previously presented as an abstract at the 13th International Symposium on Antimicrobial Agents and Resistance (ISAAR) Virtual Congress, September 9-10, 2021, Volume 58/3 PHI-008.

## Materials and methods

This prospective surveillance study was carried out in a tertiary care private medical college hospital with 2,100 beds inclusive of 140-bedded ICUs (surgical, medical, pediatric, neonatal, cardiac, respiratory, and neuro ICUs). Our ICUs are closed type, with separate specialist medical experts and supporting staff handling the different ICUs. Also, ICUs in the hospital are distinguished from the general hospital wards by a higher staff-to-patient ratio.

Our hospital is a National Accreditation Board for Hospitals & Healthcare Providers (NABH) pre-accredited hospital supported by a National Accreditation Board for Testing and Calibration Laboratories (NABL)-accredited diagnostic laboratory. Our Department of Microbiology performs culture identification and sensitivity with conventional as well as automated equipment such as the BACTEC™ Blood Culture System (BD, Franklin Lakes, NJ) and VITEK® 2 compact identification and sensitivity system (bioMérieux, Inc, Marcy-l'Étoile, France) based on Clinical and Laboratory Standards Institute (CLSI) guidelines [6].

We have a hospital infection control committee (HICC) comprising four dedicated infection control nurses (ICNs), an infection control officer, and quality champion nurses for each ward/specialized area. ICNs and quality champions were trained in batches by an infection control officer on HAI surveillance according to CDC-NHSN 2017 surveillance criteria with ICU rounds [7]. Along with this, training for hand hygiene and bundle care auditing was also given. Then, the active systematic HIC surveillance was carried out using an HAI surveillance form with bundle care auditing as a routine on a daily basis.

The present study was conducted over a period of two years from January 2019-December 2020. All patients admitted to the ICUs during the study period were included in the study. HAI surveillance form with bundle care auditing was attached to the case sheets of those patients who had at least one of the devices on them (urinary catheter, ventilator, central line). Daily appraisal forms consisting of patient days and catheter, ventilator, and central line days were filled out on a daily basis by quality champion nurses specific to that area, which were verified by ICNs.

Daily active surveillance for catheter-associated urinary tract infection (CAUTI), ventilator-associated event (VAE), and central line-associated bloodstream infection (CLABSI) was done by trained ICNs along with bundle care auditing, which was attached to the case sheets of all patients on devices. The surveillance definitions of DA-HAIs were adapted from the CDC-NHSN 2017 surveillance criteria [7].

All device-associated infections that fit the criteria were confirmed by the infection control officer and countersigned by the ICU physician. The rates of infection were calculated and compared with the benchmarks set by the INICC and NHSN [7-9]. These rates were submitted to the individual ICU and the Quality Department every month. Audit for adherence to bundle care for urinary catheter, central line, and the ventilator was performed as per the daily checklist in the surveillance form. If any deviation was found, ICU staff were educated and trained then and there itself. To overcome any form of bias, ICNs were rotated each month among different ICUs to collect the data and auditing details.

Data were analyzed at the end of every month to generate the cumulative device-associated infection (DAI) rates and DUR by using the CDC HAI rate calculation formulae. These data were compared with NHSN and INICC - India HAI rate benchmarks [7] and communicated to corresponding ICU physicians and also presented at the HICC meeting.

The formulae that were used to calculate HAI rate and DUR were as follows [7]:

DUR: number of device days/number of patient days

DAI rate: number of DAI cases [sum of ventilator-associated pneumonia (VAP), CLABSI, and CAUTI cases/total number of device days (sum of the ventilator, central line, and catheter days)] × 1,000

CAUTI rate: number of CAUTI cases/total number of catheter days × 1,000

VAE rate: number of VAE cases/total number of ventilator days × 1,000

CLABSI rate: number of CLABSI cases/total number of central line days × 1,000.

## Results

During our study period, a total of 71,877 patient days and 65,705 device days (37,979 urinary catheter days, 11,613 ventilator days, and 16,113 central line days) were noted. The overall DUR was 0.91. The DURs of the urinary catheter, ventilator, and central line were 0.53, 0.16, and 0.22 respectively.

**Figure 1:**
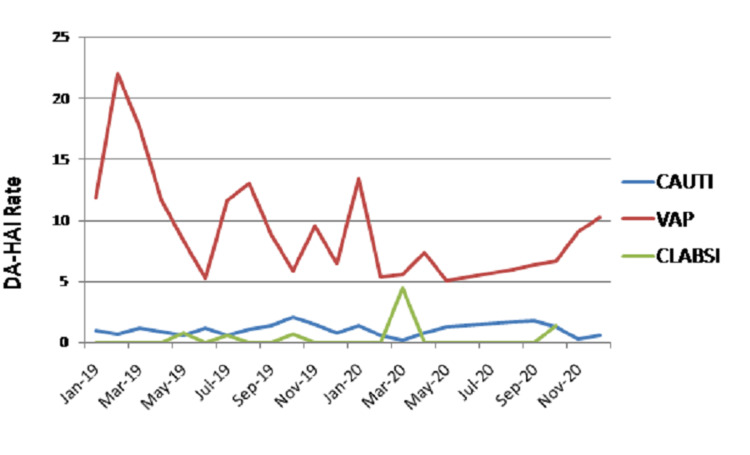
Monthly trend of DA-HAI rate in ICUs DA-HAI: device-associated hospital-acquired infections; CAUTI: catheter-associated urinary tract infection; VAE: ventilator-associated event; CLABSI: central line-associated bloodstream infection

A total of 166 DA-HAIs were noted during the study period. (CAUTI: 37; VAE: 122; CLABSI: 7). The overall DA-HAI rate was 2.52 per 1,000 device days. CAUTI, VAE, and CLABSI rates were 0.97, 10.5, and 0.43 per 1,000 device days, respectively. The analysis of DUR and DA-HAI rates (overall and for catheter, ventilator, central line) is presented in Table 1. Figure 1 depicts the monthly trend of the DA-HAI rate in ICUs.

**Table 1:** Analysis of DUR and DA-HAI rate (overall and for catheter, ventilator, central line) during January 2019-December 2020 in ICUs DUR: device utilization ratio; DA-HAI: device-associated hospital-acquired infection

Analysis (patient days: 71,877)				
	Device days	DUR	DA-HAI	DA-HAI rate/1,000 device days
Urinary catheter	37,979	0.53	37	0.97
Ventilator	11,613	0.16	122	10.5
Central line	16,113	0.22	7	0.43
Total	65,705	0.91	166	2.52

Among the ICUs, the DUR of the urinary catheter was higher in the surgical ICU (SICU: 0.75), neurotrauma ICU (NTICU: 0.74), intensive medical care unit (IMCU: 0.73), and cardiothoracic ICU (CTICU: 0.73). For ventilators, the DUR was higher in IMCU (0.44), NTICU (0.22), and for the central line, CTICU (0.72) and the specialty medical ICU (0.27) had a high DUR.

**Figure 2:**
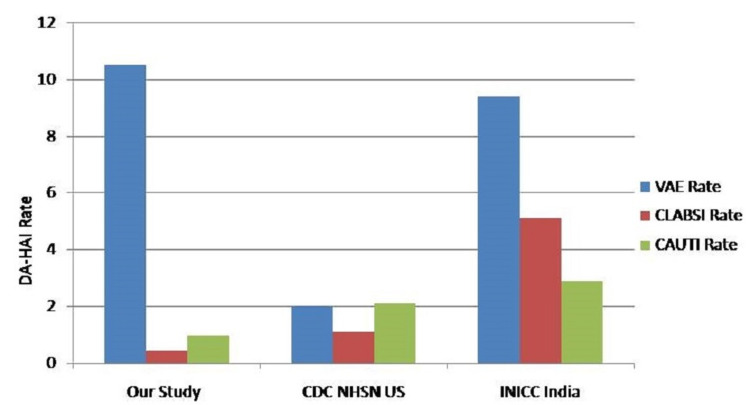
Comparison of pooled mean DAI rates of all intensive care units in our study with CDC-NHSN and INICC - India data DAI: device-associated infection; CDC-NHSN: Centers for Disease Control and Prevention's National Healthcare Safety Network; INICC: International Nosocomial Infection Control Consortium; DA-HAI: device-associated hospital-acquired infections; CAUTI: catheter-associated urinary tract infection; VAE: ventilator-associated event; CLABSI: central line-associated bloodstream infection

CAUTI rate was higher in the intensive coronary care unit (ICCU), respiratory ICU (RICU), and pediatric ICU (PICU) (2.49, 2.3, and 2.3 per 1,000 catheter days respectively). VAE rate was higher in the liver ICU (19.2), specialty medical ICU (SPL ICU: 16.6), SICU (13.4), and NTICU (12.5). CLABSI rate was 1.2 and 1 per 1,000 central line days in SPL ICU and NTICU respectively. Analyses of DU ratio, DA-HAI rate for catheter, ventilator, and the central line are presented in Table 2. Figure 2 shows the comparison of pooled mean DAI rates of all ICUs of our study with CDC-NHSN and INICC data.

**Table 2:** Analysis of DUR and DA-HAI rate for catheter, ventilator, and central line during January 2019-December 2020 in different ICUs DUR: device utilization ratio; DA-HAI: device-associated hospital-acquired infection; IMCU: intensive medical care unit; SPL ICU: specialty medical ICU; SICU: surgical ICU; HDU: high dependency unit; NTICU: neurotrauma ICU; CTICU: cardiothoracic ICU; ICCU: intensive coronary care unit; PICU: pediatric ICU; NICU: neonatal ICU; RICU: respiratory ICU

ICU	ICU (patient days)	Urinary catheter			Ventilator			Central line		
		Catheter days	DUR of catheter	CAUTI rate	Ventilator days	DUR of ventilator	VAE rate	Central line days	DUR of central line	CLABSI rate
Medical	IMCU (5,233)	3,816	0.73	1.04	2,317	0.44	9.06	964	0.18	0
	SPL ICU (15,626)	6,708	0.43	1.04	2,227	0.14	16.6	4,144	0.27	1.2
	Liver ICU (2,492)	1,279	0.51	0	260	0.10	19.2	348	0.14	0
Surgical	SICU (6,511)	4,891	0.75	0.61	1,043	0.16	13.4	917	0.14	0
	SPL SICU (3,293)	1,431	0.43	0.69	367	0.11	5.4	794	0.24	0
	Trauma ICU (2,286)	676	0.30	0	73	0.03	1.3	78	0.03	0
	HDU (2,100)	757	0.36	1.32	232	0.01	4.5	108	0.05	0
Neuro ICU	NTICU (9,202)	6,843	0.74	1.46	1,995	0.22	12.5	1,980	0.22	1.0
	Neuro ICU (2,876)	1,764	0.61	0	445	0.15	0	666	0.23	0
Cardiac ICU	CTICU (7,528)	5,489	0.73	0.18	1,335	0.18	7.49	5,420	0.72	0
	ICCU (3,425)	1,606	0.47	2.49	12	0.00	0	79	0.02	0
PICU (3,213)		432	0.13	2.3	432	0.13	4.62	99	0.03	0
NICU (4,594)		117	0.03	0	637	0.14	0	267	006	0
RICU (3,498)		2,170	0.62	2.3	2,317	0.13	8.9	249	0.07	0

Among 166 DA-HAIs reported, 182 pathogens were identified; 96.1% (175/182) isolates were Gram-negative. *Klebsiella pneumoniae* was the most common organism isolated, accounting for 37.4% of all DA-HAI cases, followed by *Acinetobacter baumanii* (30.8%). The most common organisms causing CAUTI, VAE, and CLABSI were *Klebsiella pneumoniae *(18/55, 32.7%), *Acinetobacter baumanii *(53/119, 44.5%), *Klebsiella pneumoniae *(3/8, 37.5%) respectively. Most of the Gram-negative organisms were carbapenem-resistant (153/175; 87.4%). Vancomycin resistance rate in *Enterococcus* was 28.5% (2/7). The analysis of pathogens isolated and the resistance pattern of overall and catheter-, ventilator-, central line-associated HAI are presented in Table 3.

**Table 3:** Analysis of pathogens isolated and the resistance pattern of overall and catheter-, ventilator-, and central line-associated HAI during January 2019-December 2020 in ICUs HAI: hospital-acquired infections; DAI: device-associated infections

Pathogen isolated and resistance pattern	Pathogens isolated (n=182)	
	Number of isolates from DAIs, n (%)	Resistance, n (%)
*Klebsiella pneumoniae,* carbapenem-resistant	68 (37.4)	64 (94)
*Acinetobacter baumanii, c*arbapenem-resistant	56 (30.8)	55 (98)
*Pseudomonas aeruginosa, *carbapenem-resistant	25 (13.7)	20 (80)
*Escherichia coli,* carbapenem-resistant	17 (9.3)	8 (47)
*Klebsiella aerogenes, *carbapenem-resistant	9 (4.9)	6 (66.6)
*Enterococcus* species, vancomycin-resistant	7 (3.9)	2 (28.5)

## Discussion

DU has a strong positive correlation with nosocomial infection per 1,000 patient days [10]. Our study results show a lower utilization ratio of the ventilator (0.16) and central line (0.22) compared with the ratio reported by the INICC in India (0.53 and 0.39 respectively) and other countries (0.36 and 0.54 respectively) [5,8]. As a part of our bundle care auditing, proper documentation of indication for device insertion and assessment of readiness to remove the device along with other elements were daily audited by ICNs during their active surveillance, and if any correction was needed, ICNs had a prompt discussion with the ICU in-charge to make the corrections. This may have been the major factor that influenced the lower DUR achieved in our setup. Though the DUR of the urinary catheter was 0.53, which seems higher when compared to the INICC DUR for urinary catheter (0.21), average catheter days per patient were diligently reduced to a minimum by active surveillance as the need for catheters was essential in most of the ICU patients.

CAUTI rate of 0.97 per 1,000 catheter days is much lower than the rate reported in other parts of the country (range: 1.41-9.08) and pooled INICC (2.9) and NHSN-CDC (2.1) rates, despite a higher utilization ratio of urinary catheters (0.53) [5,7,8]. The holistic approach of strict adherence to all components of bundle care with active surveillance and auditing for adherence and timely discussion with the ICU physicians led to a significant reduction in CAUTI as evidenced by our earlier study [11].

The VAE rate observed in this study (10.5 per 1,000 ventilator days) is similar to the VAP rate observed in 20 cities in India (10.4 per 1,000 ventilator days), INICC (9.4), and lower than the pooled mean VAP rate observed (16.3 per 1,000 device days) in 43 countries [5,8]. Strict adherence to infection control practices and implementation of bundle care approach led to a decrease in VAP over the period of the study. Most of the existing literature has used either the CDC’s VAP criteria or clinical pulmonary infection score with very few studies based on VAE criteria [12]. The current study used VAE surveillance, which is more objective and improves comparability. Few studies have reported difficulties in detecting traditional VAP cases by VAE surveillance [13]. This comparison was not attempted in the current study.

This study observed a CLABSI rate of 0.43 per 1,000 central line days, which is very much lower than the rate reported by other surveillance studies (range: 4.82-7.92) and INICC (5.1) and (1.1) respectively [5,8,14]. Insertion and maintenance of device care provided with the proper infection control practices over a period of time by our whole team including ICU physicians, staff nurses, and other staff led to a dramatic decrease in all DAIs.

Analysis of ICU-wise DA-HAI rate and DUR revealed that all the ICUs were on par/or even less when compared to INICC and CDC-NHSN for CAUTI and CLABSI rates. This trend applied to VAE rate as well, as most ICUs were on par with the benchmarks of INICC except in liver ICU (19.2), SPL ICU (16.6), SICU (13.4), and NTICU (12.5) where average ventilator days per patient still remained long due to the patients' critical illness. But VAE rates still showed declining trends over the period of time as shown in Figure 2.

The predominance of Gram-negative infections (*Klebsiella spp., Acinetobacter spp., Pseudomonas spp.,* and *Escherichia coli*) is similar to several other surveillance studies in India [14-16]. Organisms isolated in the present study were predominantly multidrug-resistant (94%, 98%, and 80% carbapenem resistance for *Klebsiella pneumoniae*, *Acinetobacter baumanii,* and *Pseudomonas aeruginosa* respectively). Hence, the intensivists were left with the last option of combination empirical therapy with colistin. Adding to the problem was the revised CLSI interpretation of colistin, which states that this drug is of limited clinical efficacy even for isolates with minimum inhibitory concentration (MIC) values of <2 µg/ml [17]. All these mandate enforcement of strict antibiotic stewardship programs. Most of the accreditation programs do not strictly audit antibiotic policy implementation in hospitals. In our setup, the antibiotic policy was framed, and periodic antibiotic audits were carried out by clinical pharmacists. Despite the emergence of drug-resistant microbes, compliance with hospital antibiotic policy was very low.

There have been studies on DAIs from disparate regions of the country, especially from accredited hospitals and a few government hospitals [9,12]. However, there is a paucity of data from this part of the country. This hospital-wide ICU surveillance study on DAIs has many potential areas of application. The targeted surveillance done in ICUs can be expanded to other high-risk units like postoperative wards (DUR of the urinary catheter could be high) and dialysis units (DUR of central line catheter could be high). Additionally, it can be implemented in other hospitals with microbiological facilities and staff trained in infection control activities.

Limitations

The study has a few limitations. Since it was a single-center study, the findings cannot be generalized to the wider population. The crude excess length of stay of patients with DA-HAI and overall crude excess mortality rate, when compared with patients without DA-HAI in ICUs, were not calculated.

Future direction

A few studies have reported difficulties in detecting traditional VAP cases by VAE surveillance. This comparison was not attempted in the current study. As each surveillance approach has its own advantages, in the future, both VAE and VAP surveillance can be carried out in ICU settings to see if there is any difference in population characteristics. 

Studies related to the economic impact of DA-HAI on the patients (in terms of excess stay, antibiotic usage cost, etc.) as well as the psychological impact will be very useful to emphasize the importance of surveillance to prevent HAIs.

## Conclusions

Active surveillance with bundle care auditing and timely discussion with the ICU physicians by a trained team led to a significant change in terms of low DUR, which in turn resulted in lesser/on par rates against the benchmarks of INICC and CDC-NHSN in terms of CAUTI, VAE, and CLABSI rates per 1,000 device days in almost all ICUs of our tertiary care unit. Gram-negative isolates were the predominant pathogens isolated (96.4%) and of these, 87.4% had carbapenem resistance, leaving physicians with few antibiotics of choice to save the patients' lives. Proper active surveillance with bundle care and training promptly by ICNs made a huge difference in all DA-HAI rates, especially VAE, which decreased to 10.5 from 23.6 per 1,000 ventilator days.

Thus, in any hospital, sustained active surveillance of HAI and bundle care auditing by a trained infection prevention team with a stringent antibiotic policy are the need of the hour to combat DAIs.
